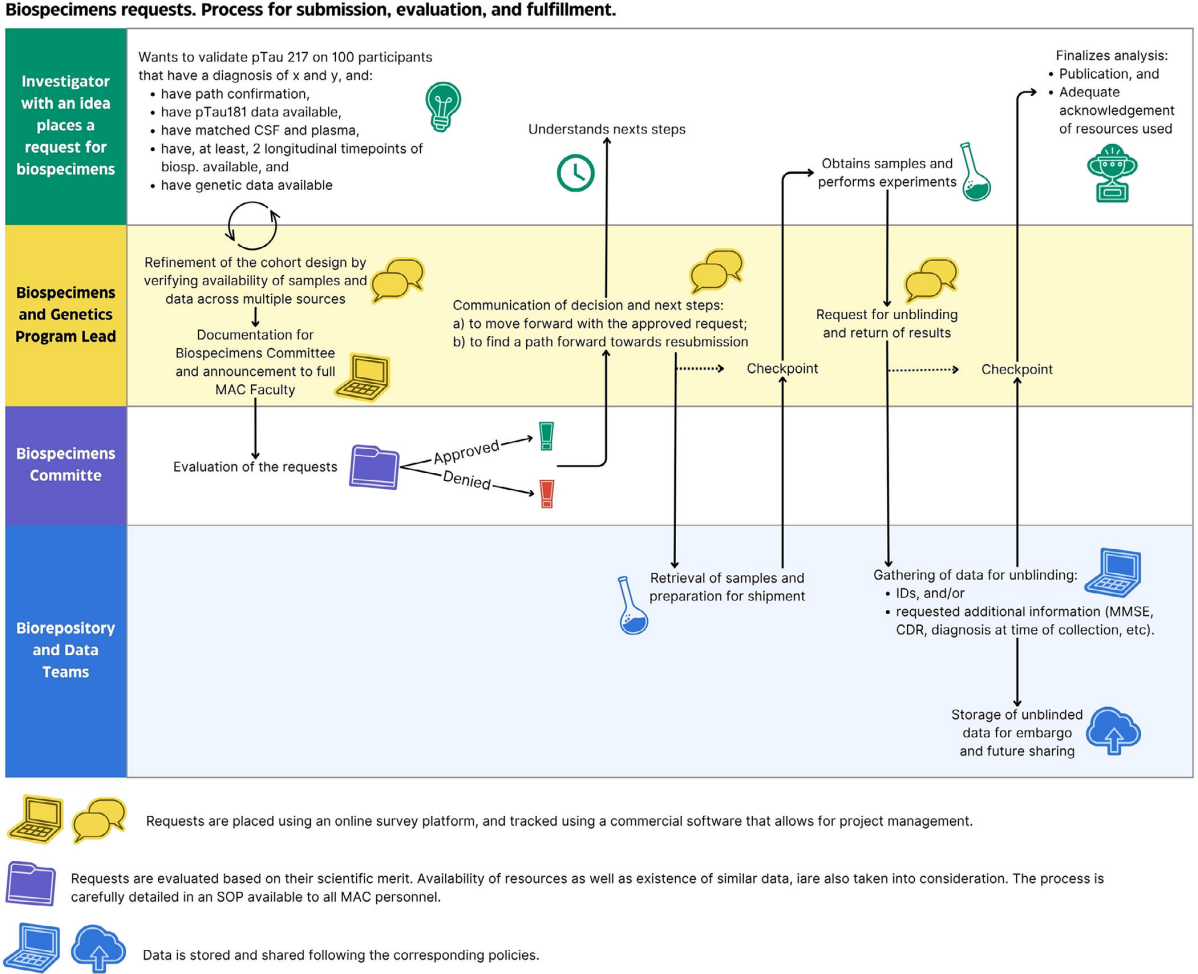# Fluid biospecimens and genetic data collection and dissemination: new pipelines to serve the dementia research community from the UCSF Memory and Aging Center

**DOI:** 10.1002/alz.091555

**Published:** 2025-01-09

**Authors:** Argentina Lario Lago, Julia D Webb, Taylor M Young, Rose George, Karen Smith, Ana Tyler, Eliana Marisa Ramos, Hilary W. Heuer, Mary Koestler, Lawren VandeVrede, Renaud La Joie, Gil D. Rabinovici, Bruce L. Miller, Julio C. Rojas, Kaitlin B. Casaletto, Jennifer S. Yokoyama, Adam L. Boxer

**Affiliations:** ^1^ Memory and Aging Center, UCSF Weill Institute for Neurosciences, University of California San Francisco, San Francisco, CA USA; ^2^ Memory and Aging Center, UCSF Weill Institute for Neurosciences, University of California, San Francisco, San Francisco, CA USA; ^3^ University of California, Los Angeles, Los Angeles, CA USA; ^4^ University of California San Francisco (UCSF), San Francisco, CA USA; ^5^ Memory and Aging Center, Weill Institute for Neurosciences, University of California, San Francisco, San Francisco, CA USA; ^6^ Memory and Aging Center, UCSF Weill Institute for Neurosciences, San Francisco, CA USA

## Abstract

**Background:**

Collection, storage, and distribution of human fluid biospecimens in a scientifically rigorous manner is challenging. It requires physical space availability and robust infrastructure. Nonetheless, it is key to contribute to research in Alzheimer’s Disease and Related Disorders, including Frontotemporal Dementia (FTD). These specimens facilitate fluid biomarker discovery and validation studies. At the UCSF Memory and Aging center (MAC) we support 31 protocols, from both clinical and observational studies, including international ones (e.g. ALLFTD). Besides specimens, our Biospecimens and Genetics Program (BsGP) stores and shares fluid biomarker and genetic data associated with specimens collected under such studies. Here, we aim to report results of a single center’s operational efforts to create a platform for biospecimens and biospecimens‐related data management that functions seamlessly allowing for collaborative research.

**Method:**

Over the past three years, we developed infrastructure to support biospecimen research, both locally and internationally. Our program is led by a PhD neuroscientist and supported by four faculty members. We have a dedicated data manager, an administrative coordinator, and five members in our processing lab. We doubled our laboratory equipment (acquired two centrifuges) and implemented a superior version of our Laboratory Information Management System. We maintained and expanded partnerships with entities like UCSF‐IHG, UCLA, NCRAD, ATRI. We strengthened our technological infrastructure by hiring a dedicated programmer. We transitioned towards a de‐centralized biobanking strategy.

**Result:**

During 2023, the BsGP supported 16 investigators who submitted 20 genetic data requests. We supported our Fibroblast, Genetic, and Neuropathology Cores by providing quarterly customized genetic datasets. Our BsGP Committee reviewed 31 biospecimens requests, including 11 from non‐UCSF affiliated investigators (from academic institutions and industry). They all required heavily leveraging internal resources to provide additional clinical and biomarker data. We began to build a bespoke bioinformatics solution for our program: SYNC. SYNC will facilitate tracking and searching for biospecimens (and associated fluid biomarker and genetic data) easily, so that accessing existing resources is not a roadblock for research.

**Conclusion:**

The MAC BsGP can serve as a model for centers interested in improving their biospecimen research programs, which requires dedicated investments in infrastructure, especially hiring and supporting qualified personnel.